# Salivary Gland Choristoma of the Larynx

**DOI:** 10.4274/balkanmedj.2016.0657

**Published:** 2017-05-15

**Authors:** Rukiye Yılmaz, Recep Bedir, İbrahim Şehitoğlu, Engin Dursun

**Affiliations:** 1 Department of Pathology, Recep Tayyip Erdoğan University School of Medicine, Rize, Turkey; 2 Department of Otorhinolaryngology, Recep Tayyip Erdoğan University School of Medicine, Rize, Turkey

## To The Editor,

**Figure 1:**
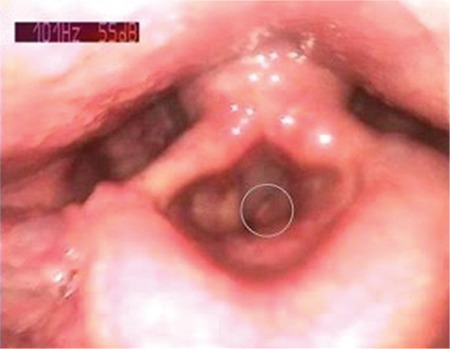
Laryngoscopy of the lesion in the left vocal cord (marked with a circle).

**Figure 2:**
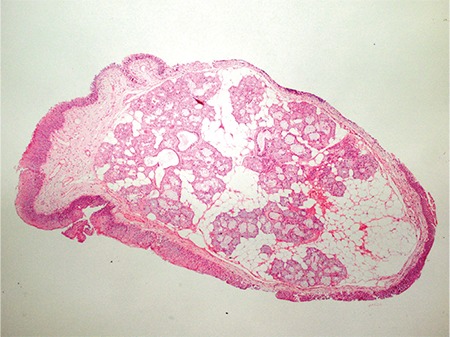
Normal salivary gland acini were seen on the vocal cord (H&E X40).

**Figure 3:**
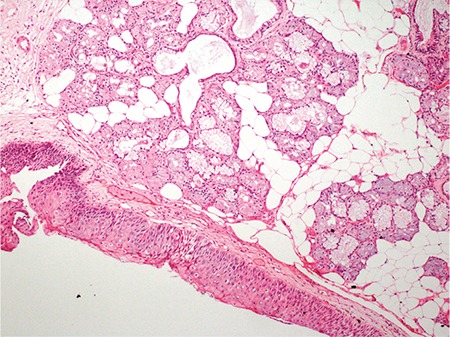
The lesion composed of benign salivary gland acini and lobulus (H&E X200).

Choristoma is defined as the presence of histologically normal cells in abnormal locations due to defects during embryological development (1). The criteria for the diagnosis of choristoma are a tumour-like growth, an ectopic tissue with a normal pattern and without neoplastic features histologically, and a mislocated tissue topographically. It is different from hamartoma because the hamartoma appears in normal locations. Laryngeal choristomas are rare lesions and are usually relevant to glial or thyroid tissues (2). Salivary gland choristomas (SGC) in the cheek, middle ear, neck, jaw, thyroid gland, pituitary gland, mediastinal lymph nodes, breast, anterior chest wall, oesophagus, duodenum, jejunum, rectum and amygdala have been reported (3). However, SGC of the larynx is very rare (4). A 43 year-old male patient was referred to our hospital with the complaint of hoarseness and productive coughing for six months. The patient had been smoking cigarettes for 15 years (20 cigarettes per day), but did not consume alcohol. There was neither intubation history nor any other previous history of laryngeal trauma in the patient's past. Written informed consent was obtained from the patient. Laryngoscopic examination was performed, which revealed the presence of a lesion on the anterior region of the left vocal cord. The lesion was 0.5 cm in size and had a polypoid appearance (Figure 1). The lesion was completely removed by direct suspension laryngoscopy. Macroscopically, the lesion was about 0.5 cm in diameter, soft and polypoid. Microscopically, the mucosa was intact and there were no dysplasia, mitoses or any other signs of malignancy in the squamous epithelium. A choristoma-heterotopic salivary gland tissue was found under the normal epithelium (Figure 2). The lesion was composed of salivary gland acini (Figure 3). With these morphological findings, the case was reported as a SGC.

SGCs are infrequent benign lesions. Only two cases have been described in the literature to date (2,4). The pathogenesis of this entity is still uncertain and is related to developmental anomalies. The differential diagnoses of these masses in the larynx comprise benign lesions such as laryngeal cyst, laryngeal nodules, contact ulcers, squamous papilloma, amyloidosis or granulomatous lesions such as Wegener's granulomatosus, sarcoidosis and tuberculosis (2). These conditions can be excluded by careful histopathological examination. Simple excision is sufficient for the treatment of these lesions.

SGC should also be differentiated from some infrequent malignant lesions. Some of these lesions are primary laryngeal adenocarcinomas, metastatic adenocarcinoma, salivary glandular tumours like acinic cell carcinoma, mucoepidermoid carcinoma or adenoid cystic carcinoma of the larynx. These uncommon tumours are also located in other areas (5). Surgical excision is sufficient for treatment. Histopathological findings are useful for distinguishing this rare lesion from rare malignant tumours. In our case, there was no histological evidence of malignancy, necrosis, atypical and typical mitotic cells and pleomorphism. In conclusion, awareness of this rare entity is essential to avoid misdiagnosis.
